# Crystallization of Polymers Investigated by Temperature-Modulated DSC

**DOI:** 10.3390/ma10040442

**Published:** 2017-04-24

**Authors:** Maria Cristina Righetti

**Affiliations:** National Research Council of Italy—Institute for Chemical and Physical Processes (CNR-IPCF), Via Moruzzi 1, 56124 Pisa, Italy; cristina.righetti@pi.ipcf.cnr.it; Tel.: +39-050-315-2068

**Keywords:** polymer, crystallization, differential scanning calorimetry, temperature-modulated differential scanning calorimetry, reversing melting, reversible melting, crystalline fraction, mobile amorphous fraction, rigid amorphous fraction

## Abstract

The aim of this review is to summarize studies conducted by temperature-modulated differential scanning calorimetry (TMDSC) on polymer crystallization. This technique can provide several advantages for the analysis of polymers with respect to conventional differential scanning calorimetry. Crystallizations conducted by TMDSC in different experimental conditions are analysed and discussed, in order to illustrate the type of information that can be deduced. Isothermal and non-isothermal crystallizations upon heating and cooling are examined separately, together with the relevant mathematical treatments that allow the evolution of the crystalline, mobile amorphous and rigid amorphous fractions to be determined. The phenomena of ‘reversing’ and ‘reversible‘ melting are explicated through the analysis of the thermal response of various semi-crystalline polymers to temperature modulation.

## 1. Introduction

Temperature-modulated differential scanning calorimetry (TMDSC), in which a periodic temperature perturbation is superimposed on a linear heating or cooling or takes place around a fixed temperature, has become, in recent years, a technique extensively used for the characterization of polymeric materials [1,2]. TMDSC, which was introduced commercially in the 1990s [3], has been proven to provide several advantages for the analysis of polymers with respect to standard differential scanning calorimetry (DSC), allowing the clarification of important aspects of polymer transitions.

As regards crystallization and melting, TMDSC has allowed the reversibility of latent heats contribution to be proven also in the case of polymers. In principle, melting and crystallization of polymers should be irreversible processes, as crystallization needs considerable undercooling, and the temperature modulation is generally only fractions of degrees or few degrees [1,2]. Actually, it has been demonstrated that melting and crystallization of polymers can also be reversed by small temperature modulations, and, in some cases, exhibit reversible features [1,2]. For this reason, the term ‘reversing melting’ was introduced [4,5]. Reversing melting identifies a situation in which the modulation amplitude connects the temperature intervals in which melting and crystallization take place. Only processes that are reversing on the timescale of the temperature modulation contribute to the reversing signal. This seems in contrast with the requirement of an undercooling, which is generally of the order of 10 K for the crystallization of high molar mass polymers [2,5]. The phenomenon has been imagined and rationalized by considering the particular organization of the chains in a semi-crystalline polymer. A macromolecule, due to its length, may be partially in the crystal and partially in the melt. Detachment of segments and subsequent attachment can take place without the need of molecular nucleation, with the result that the difference between the crystallization and melting temperatures becomes smaller [2,5]. This could be the mechanism of reversing melting in polymers that do not exhibit sliding diffusion. In this case, the phenomenon could take place at the lateral surfaces [2,6,7], whereas polymers characterized by sliding diffusion could undergo reversing melting at the fold surfaces, as changes in the fold-length can occur without melting [8,9].

The specific heat capacity that is derived from TMDSC experiments, which is connected to the heat flow that follows temperature modulation, is called reversing specific heat capacity (*c_p,rev_*). According to the mathematical treatment generally applied to TMDSC data, the modulated heat flow rate curve is expressed as a Fourier series [10,11,12,13]. If linear condition is fulfilled (i.e., if the perturbation amplitude is doubled, the resulting response amplitude is doubled too) and stationarity holds (i.e., the relationship between the perturbation and the response does not change during one modulation period) [14], the first harmonic is able to describe the response of the sample to modulation. Thus, *c_p,rev_* can be obtained from the ratio between the amplitudes of the first harmonic of the modulated heat flow rate (*A_HF_*) and the temperature modulation (*A_T_*) [10,11,12,13]:

(1) $$c_{p,rev}(\omega ,t,T)=\frac{A_{HF}(t,T)\;}{A_{T}(t,T)}\frac{K(\omega )}{m\omega }$$

where ω is the frequency of temperature modulation (ω = 2π/*p* with *p* representing the modulation period), *m* the mass of the sample and *K*(ω) the frequency-dependent calibration factor. The most commonly used temperature modulations have sinusoidal shape or sawtooth profile: in the latter case, the temperature is also described with a Fourier series, and *A_T_* corresponds to the amplitude of the first harmonic [13]. The sawtooth temperature modulation appears more appropriate for the investigation of polymer melting and crystallization, if steady state is reached in each semi-period [7]. Appropriate measurement conditions must be chosen in order to obtain correct *c_p,rev_* data. Linear and stationary conditions are generally achieved by using temperature perturbation as small as possible: the modulation amplitude must be small enough to avoid important changes of specific heat capacity during a single period, and the average scanning rate (*q*) and the modulation period also have to be low, so that the average temperature change during one modulation period is smaller than the temperature amplitude [14,15]. The correct operative conditions depend on the sample and thermal transition under investigation. To improve linearity in the melting region, heating only condition has to be used (ωA*_T_* < *q*) [14]. This requisite, which hinders recrystallization, can conversely be inappropriate for the study of a crystallization process. Non-linearity and non-stationarity cause the presence of higher harmonics in the heat flow rate signal, which influences the accuracy of *c_p,rev_* determined by Equation (1). Therefore, correctness of the reversing specific heat capacity should be verified by checking the potential presence of higher even harmonics in the measured modulated heat flow rate [14,15]. With the conventional TMDSC instruments, the modulation period ranges approximately from 40 s to 200 s, the amplitude is comprised between 0.1 K and 2 K, and the average scanning rate is not higher than 3 K min^−1^ [1,2]. Modulation of temperature can be obtained also by combining short heating or cooling steps with isotherms [1,16]. The mathematical treatment of these ’step-scan’ experiments is different, but the information derived on the reversibility of the processes is similar [1,16]. The temperature-time profile in the temperature-modulated experiment can however be any periodic function. If non-harmonic temperature perturbations are applied, the response is a multi-frequency signal. Simultaneous multi-frequency measurements can thus be performed [17,18]. Temperature-modulated measurements can also be performed by using fast scanning nano-calorimeters [19,20], which allow the accessible frequency range to be extended to higher frequencies (0.1–100 Hz) [21]. In this review, however, only experiments carried out with conventional TMDSC instruments (sinusoidal or sawtooth temperature-modulation) will be reported and discussed. 

In the crystallization and melting region, the reversing specific heat capacity is generally a superposition of the baseline specific heat capacity (*c_p,base_*), which corresponds to the specific heat capacity needed to increase the temperature of the sample without changing crystallinity [22], and an excess specific heat capacity (*c_p,exc_*), which in turn originates from the latent heats of the melting and crystallization processes that occur during the two modulation semi-periods, respectively [1,2]. When *c_p,exc_* is zero, the reversing specific heat capacity corresponds to *c_p,base_*, which means that crystallization or melting take place irreversibly with the same rate during the two semi-periods. Conversely, when *c_p,exc_* is higher than zero, reversing processes, i.e. melting and crystallization, occur in the two semi-periods, respectively. If the exothermic and endothermic contributions are symmetric, the reversing-specific heat capacity can be considered reversible within the chosen modulation amplitude and frequency. Conversely, when the exothermic and endothermic latent heats are asymmetric, *c_p,rev_* depends on the progress of the phase transition, which can occur partially or completely in an irreversible way. For this reason, *c_p,rev_* is designated as ‘reversing’, in order to distinguish it from a true reversible situation. As better detailed in Section 3, after sufficiently long time in quasi-isothermal conditions, the irreversible events are completed, and the reversing-specific heat capacity tends to the reversible *c_p,rev_* value [12,23]. 

The crystallization process of polymers has been widely investigated by TMDSC [7,22,24,25,26,27,28,29,30,31,32,33,34,35,36,37,38]. An accurate investigation of the crystallization process is a fundamental step for a full comprehension of the polymer structure, which in turn strongly influences the material properties. 

In this review, studies conducted by TMDSC on various polymers in different crystallization conditions will be reported and discussed, with the aim of illustrating the type of information that can be deduced. Crystallizations occurring in different experimental conditions will be examined and discussed in different sections. The novelty of the results that this technique can provide about the crystallization process of polymers will be thus identified and highlighted.

## 2. Non-Isothermal Crystallization and Stepwise Quasi-Isothermal Crystallization upon Heating

Polymer crystallization that takes place above the glass transition temperature (*T_g_*) upon heating, in the presence of nuclei grown during the previous cooling step or during a permanence of the sample at temperatures below *T_g_*, is the so-called ‘cold-crystallization’ process [39]. This type of crystallization occurs at temperatures well below the melting region, far from equilibrium, and due to high nucleation density, it is generally fast. For these reasons this process may not be modulated, and commonly it is observed as a fully irreversible phenomenon in TMDSC curves [1,2].

**Figure 1:**
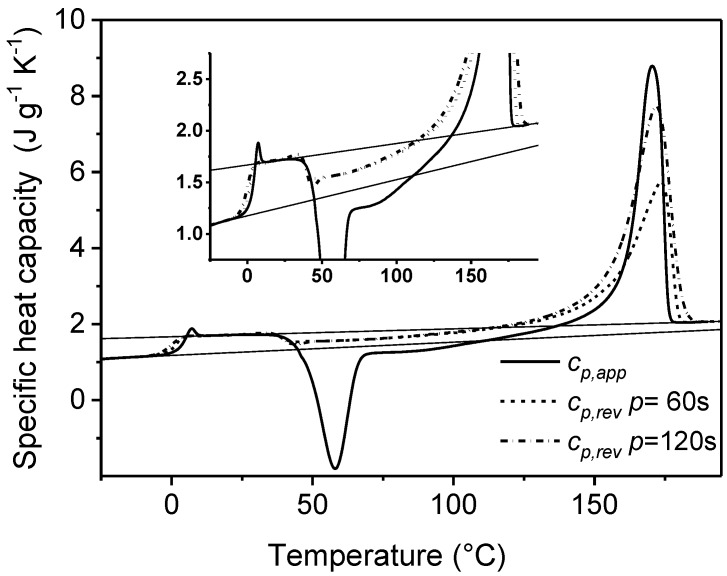
Apparent specific heat capacity (*c_p,app_*) by standard differential scanning calorimetry (DSC) and reversing-specific heat capacity (*c_p,rev_*) of poly(3-hydroxybutyrate) (PHB) measured after cooling from the melt at 200 K min^−1^ at modulation periods *p* = 60 s and 120 s (*A_T_* = 1.0 K, *q* = 2 K min^−1^). The thin solid lines are the solid- and liquid-specific heat capacities, as taken from [22]. The inset is an enlargement of the Figure in the cold crystallization region. (Reprinted (adapted) with permission from [35]. Copyright (2012) American Chemical Society).

An example is reported in Figure 1, which shows the comparison between the *c_p,app_* curve from standard DSC and the *c_p,rev_* curves at two different frequencies of temperature modulation for an initially amorphous poly(3-hydroxybutyrate) (PHB) sample. Above the glass transition temperature, which is located around 0 °C, in the temperature range 25–45 °C, the *c_p,rev_* curves overlap the apparent specific heat capacity (*c_p,app_*) data, as well as the thermodynamic specific heat capacity of liquid PHB, attesting that in this interval no processes involving latent heat take place. Around 30–45 °C, simultaneously with the appearance of the cold crystallization peak in the *c_p,app_* curve, the *c_p,rev_* value suddenly decreases. The *c_p,rev_* curves overlap in the interval 45–75 °C, which proves that the reversing specific heat capacity is independent of the modulation frequency in this temperature range, and that no reversing latent heat is exchanged. This means that, in this temperature range, crystallization and melting do not take place consecutively in the two semi-periods, respectively, and that the *c_p,rev_* curve corresponds to the baseline specific heat capacity. In fact, the general rule for the interpretation of the TMDSC curves is that when crystallization and melting latent heats do not contribute to the reversing specific heat capacity, i.e., when the excess specific heat capacity is zero, *c_p,rev_* is independent of the modulation frequency and corresponds to the baseline specific heat capacity of the crystallization process. The evolution of crystallinity can be thus followed by changes in *c_p,rev_* [22,31], and the rapid decrease in *c_p,rev_* around 30–45 °C has to be ascribed to an increased solid fraction of PHB, which develops during an irreversible process that occurs continuously both in the heating and cooling semi-periods with the same rate. In contrast, above 75 °C, the frequency dependence of the *c_p,rev_* curves attestes that thermal processes involving reversing exchanges of latent heat, typical of polymer melting, take place in this temperature range [1,2].

**Figure 2:**
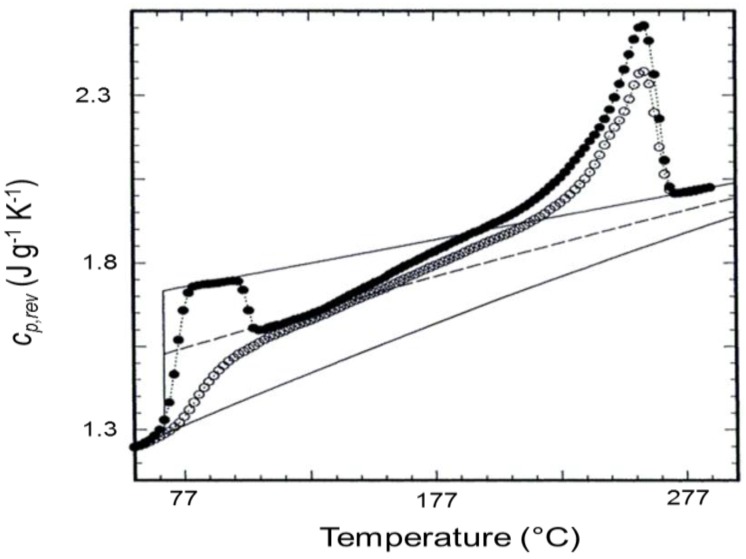
Reversing-specific heat capacity (*c_p,rev_*) of a melt-quenched amorphous poly(ethylene terephthalate) (PET) sample (filled circles) and a melt-crystallized PET sample (open circles) with crystallinity degree of 44%, measured by quasi-isothermal temperature-modulated differential scanning calorimetry (TMDSC) upon stepwise heating (*p* = 60 s and *A_T_* = 1.0 K, oscillation time around each temperature: 20 min). The thin solid lines are the solid and liquid specific heat capacities, as taken from [40]. The broken line is the calculated specific heat capacity for a 40% crystalline PET. (Reprinted (adapted) with permission from [5]. Copyright (1997) American Chemical Society).

A second example of cold crystallization studied by TMDSC is illustrated in Figure 2. In this case, the cold crystallization was monitored during heating through a stepwise ‘quasi-isothermal’ method. Every 2 K, a quasi-isothermal experiment of 20 min was performed, by oscillating the temperature around a fixed value. Also in Figure 2, as in Figure 1, the *c_p,rev_* curve of a melt-quenched, amorphous sample of poly(ethylene terephthalate) (PET) exhibits a sharp decrease around 100 °C, simultaneously with the cold-crystallization process. After cold crystallization, the crystallinity amount, determined by standard DSC, was 40%. For a comparison, Figure 2 also shows the *c_p,rev_* curve of a semicrystalline PET sample with similar crystallinity degree, obtained with the same TMDSC experimental procedure.

## 3. Quasi-Isothermal Crystallization

The isothermal crystallization of several polymers has been investigated by TMDSC [9,22,24,25,26,27,28,29,30,31,32,34,36,38]. Crystallization is monitored with this technique by oscillating the temperature around a predefined crystallization temperature, with amplitude ranging from fractions of degrees to few degrees [1,2]. 

For polycarbonate (PC) and PHB [22,29,31], no contribution of latent heat to the measured reversing-specific heat capacity was detected during crystallization at low temperature, i.e., slightly above the respective glass transition temperatures. 

**Figure 3:**
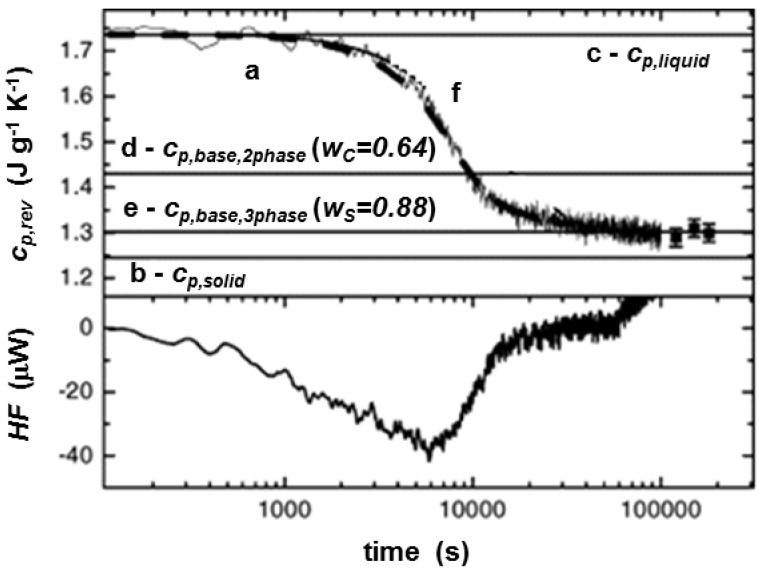
Time evolution of reversing specific heat capacity (*c_p,rev_*) during quasi-isothermal crystallization of PHB at 23 °C (*p* = 100 s and *A_T_*= 0.4 K), curve a. Curves b and c correspond to the solid and liquid specific heat capacities (*c_p,solid_* and *c_p,liquid_*), respectively, as taken from [22]. Curve d was estimated from a two-phase model, Equation (3), and curve e from a three-phase model, Equation (5). The squares represent measurements at modulation periods ranging from 240 to 1200 s. Curve f (thick dashed line) is the expected *c_p,rev_* curve from model calculations, see text. At the bottom, *HF* is the average heat flow rate (Reprinted (adapted) with permission from [31]. Copyright (2003) Elsevier).

Figure 3 shows the time evolution of the reversing-specific heat capacity of PHB during quasi-isothermal crystallization at 23 °C [22,31]. At very low crystallization temperature, the dynamics of the crystallization is slow, and the conditions of linearity and stationarity of the heat flow signal are better satisfied [14,15]. The reversing specific heat capacity decays with time from the liquid specific heat capacity value at the crystallization temperature, to an approximately constant final value. The frequency independence was checked by measuring *c_p,rev_* at the end of crystallization with different modulation periods. 

The final crystalline weight fraction (*w_C_*), equal to 0.64, was obtained from integration of the complete average heat flow rate curve, divided by the enthalpy of fusion of the 100% crystalline polymer at the crystallization temperature, $\Delta h_{m}^{o}\left(T_{c}\right)$, according to Equation (2) [24]:

(2) $$w_{C}\left(t,T_{c}\right)=\frac{\int_{0}^{t}HF\left(t\prime ,T_{c}\right)dt\prime }{\Delta h_{m}^{o}\left(T_{c}\right)}$$

At the end of the crystallization, the measured *c_p,rev_* was smaller than the value of the two-phase baseline, calculated according to the following equation [22,31]:

(3) $$c_{p,base,2phase}=w_{C}c_{p,solid}+\left(1-w_{C}\right)c_{p,liquid}$$

where *c_p,solid_* and *c_p,liquid_* are the solid and liquid specific heat capacities of PHB at the crystallization temperature. This indicated that an additional solid fraction developed during the crystallization process. This solid non-crystalline fraction is the ‘rigid amorphous’ (RA) fraction [2]. As the length of the polymer molecules is much higher than the dimensions of the crystalline phase, at least in one direction, a frequent crossing of chains occurs at the amorphous/crystal interface. This interface, with nano-metric dimensions, constituted by amorphous chain segments with mobility hindered by the near crystalline structures, is the RA fraction. The mobility of the RA fraction is lower than that of the unconstrained amorphous phase, which, for this reason, is usually named ‘mobile amorphous’ (MA) fraction.

The total solid fraction (*w_S_*), after completion of crystallization, was estimated at *T_g_* from the relationship [22]:

(4) $$w_{S}=w_{C}+w_{RA}=\left(1-w_{MA}\right)=1-\frac{\Delta c_{p}}{\Delta c_{p,a}}$$

where Δ*c_p_* and Δ*c_p,a_* are the specific heat capacity increments at *T_g_* for the semi-crystalline and the fully amorphous polymer, respectively. The *w_S_* value at *T_g_* was 0.88, which means that the RA weight fraction (*w_RA_*) was 0.24. The three-phase baseline was thus calculated [22,31]:

(5) $$c_{p,base,3phase}=w_{C}c_{p,solid}+w_{RA}c_{p,solid}+w_{MA}c_{p,liquid}$$

being *c_p,solid_* = *c_p,crystal_* = *c_p,rigid amorphous_* = *c_p glass_* [41]. For most polymers, the thermodynamic *c_p,solid_* and *c_p,liquid_* are available from [40].

The proof that the evolution of the *c_p,rev_* curve corresponds to the evolution of the baseline specific heat capacity during the entire crystallization process was achieved by simulating the time dependence of *c_p,base_* according to the following equation [31]:

(6) $$c_{p,base}=c_{p,liquid}-\frac{w_{C}\left(t\right)}{w_{C}\left(\infty \right)}\left(c_{p,liquid}-c_{p,base}\left(\infty \right)\right)$$

where *c_p,base_*(∞) was assumed equal to [*w_S_*(∞) *c_p,solid_* + (1 − *w_S_*(∞)) *c_p,liquid_*] with *w_S_*(∞) = 0.88. The dashed line in Figure 3 shows that the agreement between the experiment and the predicted data is perfect. This proves that the rigid amorphous fraction in PHB at 23 °C is formed during the whole crystallization process, simultaneously with the crystal growth. 

The rigid amorphous fraction evolution does not always parallel the crystalline development. In order to highlight how the crystallization temperature can influence the RA fraction development, quasi-isothermal crystallization of poly(l-lactic acid) (PLLA) was performed at different temperatures. Also, PLLA is a polymer that, like PC and PHB, does not exhibit dependence of the reversing-specific heat capacity on the modulation frequency [34,38]. The sample was crystallized at *T_c_* = 90 °C, a temperature at which the slightly distorted and disordered α′-form grows, and at *T_c_* = 130 °C and 135 °C, which allows crystallization of the more stable α-form [42,43,44].

**Figure 4:**
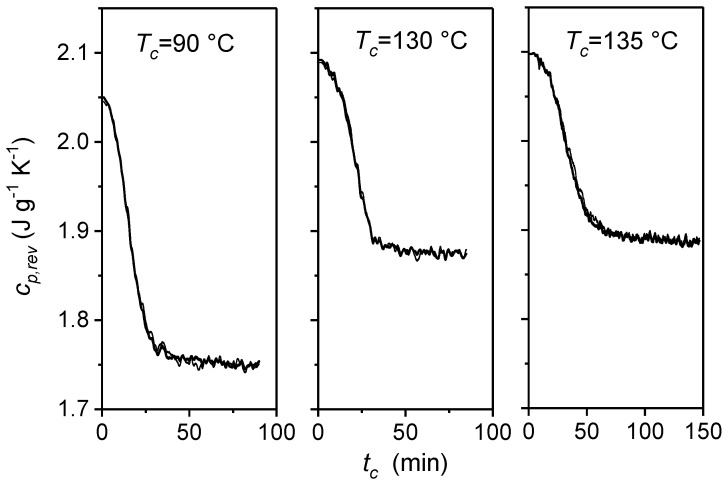
Crystallization time (*t_c_*) evolution of the reversing specific heat capacities (*c_p,rev_*) of poly(l-lactic acid) (PLLA) during quasi-isothermal crystallization from the melt at *T_c_* = 90 °C, 130 °C, and 135 °C, respectively (*p* = 60 s: thick line, *p* = 120 s: thin line, *A_T_* = 0.4 K). (Reprinted (adapted) with permission from [34]. Copyright (2011) Elsevier).

The time evolution of the reversing specific heat capacity (*c_p,rev_*) of PLLA at two different modulation periods (*p* = 60 s and 120 s), during quasi-isothermal crystallization at various *T_c_*s, is displayed in Figure 4. The *c_p,rev_* curves at *p* = 60 s and 120 s fully overlap, within the experimental noise, at the three *T_c_s* investigated, which proves that *c_p,rev_* corresponds to the baseline specific heat capacity of the crystallization process and that crystallization of PLLA occurs irreversibly in a wide temperature range. Figure 4 shows the decay of the reversing specific heat capacity curves during crystallization from the liquid specific heat capacity values at the respective *T_c_s*, to an approximately constant final value. From these *c_p,rev_* curves, the time evolution of the mobile amorphous weight fraction (*w_MA_*) during quasi-isothermal crystallization was calculated according to the following equation [31]:

(7) $$w_{MA}\left(t,T\right)=\frac{c_{p,rev}\left(t,T\right)-c_{p,solid}\left(T\right)}{c_{p,liquid}\left(t,T\right)-c_{p,solid}\left(T\right)}$$

For PLLA, *c_p,solid_* and *c_p,liquid_* were taken from [45]. 

**Figure 5:**
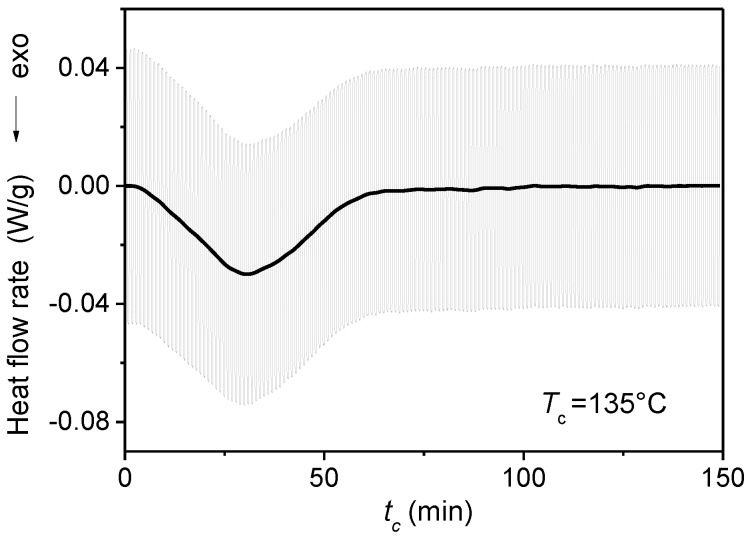
Crystallization time (*t_c_*) evolution of the modulated heat flow rate (*HF_mod_*: grey line) and average heat flow rate (*HF*: black line) during quasi-isothermal crystallization of PLLA (*p* = 60 s, *A_T_* = 0.4 K) at *T_c_* = 135 °C. (Reprinted (adapted) with permission from [34]. Copyright (2011) Elsevier).

From the average heat flow rate, the growth kinetics of the crystalline weight fraction (*w_C_*) was obtained. An example is shown in Figure 5. From the partial areas of the average heat flow rate (*HF*), which is drawn superimposed on the experimental modulated *HF_mod_* signal at *T_c_* = 135 °C, the *w_C_* curve was derived according to Equation (2). For crystallization at *T_c_* = 90 °C, the $\Delta h_{m}^{o}\left(T_{c}\right)$ of the α′-form was used, whereas for crystallization at *T_c_* = 130 °C, and 135 °C, the $\Delta h_{m}^{o}\left(T_{c}\right)$ values referring to the α-form were applied [46]. Finally, the rigid amorphous weight fraction (*w_RA_*) evolution was determined by difference, being (*w_C_* + *w_MA_* + *w_RA_*) =1. 

**Figure 6:**
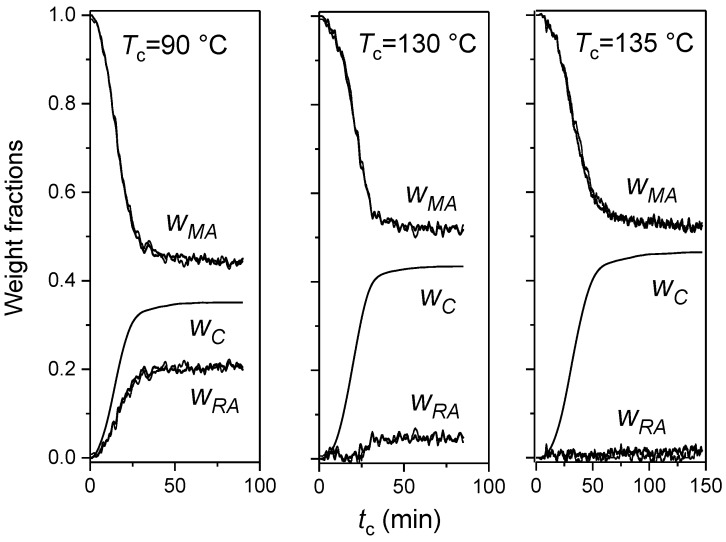
Time evolution of crystalline (*w_C_*), mobile amorphous (*w_MA_*) and rigid amorphous (*w_RA_*) weight fractions during quasi-isothermal crystallization from the melt of PLLA at *T_c_* = 90 °C, 130 °C, and 135 °C (*p* = 60 s: thick line, *p* = 120 s: thin line, *A_T_* = 0.4 K). Estimated errors: ±0.02 for *w_C_* and *w_MA_*, ±0.04 for *w_RA_*. (Reprinted (adapted) with permission from [34]. Copyright (2011) Elsevier).

The time evolution of the crystalline, mobile amorphous and rigid amorphous weight fractions of PLLA during quasi-isothermal TMDSC analyses is illustrated in Figure 6. As expected, the final crystalline degree increases with the crystallization temperature. Also, the *w_RA_* evolution is found dependent on the crystallization temperature. The rigid amorphous fraction grows in parallel with *w_C_* from the beginning of the crystallization process during the entire solidification process at low *T_c_*. During crystallization at intermediate temperature (*T_c_* = 130 °C), the RA fraction starts to appear at longer times, approximately simultaneously with the secondary crystallization process, which generally takes place in geometrically restricted regions, after spherulite impingement, approximately connected with the inflection point of the *w_C_* curve [47]. At higher *T*_c_*s*, RA fraction does not form. 

These trends prove the existence of a crystallization temperature limit for the formation of RA fraction in PLLA, as already proven for other different semi-crystalline polymers [34,36,37,48,49]. The dissimilar evolution of the RA fraction at different *T_c_s* can originate from different mobility of the chains and different crystalline organizations and morphologies [34,36,37,48,49]. At high crystallization temperature, the macromolecules have high mobility, which facilitates the organization of the polymeric segments into ordered crystal structures, with minor or no fraction of amorphous segments at the amorphous/crystal interface subjected to geometrical constraints. At high *T_c_*, the crystallization rate is lower, and this condition can also allow the growth of regular lamellae characterized by adjacent regular re-entry folding. Conversely, the low chain mobility at low crystallization temperatures implies a more difficult organization of the entangled chain segments into ordered crystal structures, which can lead to the development of irregular chain cluster structures, and large rigid amorphous fraction at the amorphous/crystal boundaries. 

**Figure 7:**
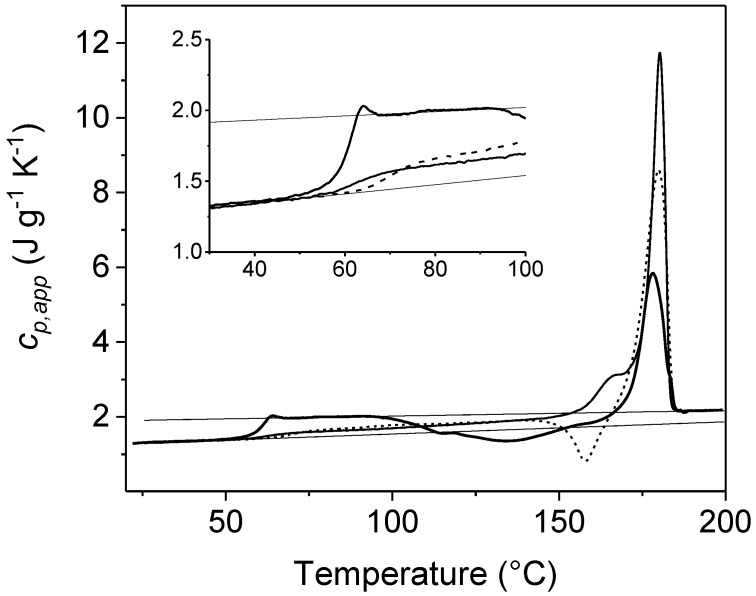
Apparent specific heat capacity (*c_p,app_*) of an amorphous PLLA sample (thickest solid line) and after complete isothermal crystallization at *T_c_* = 85 °C (dotted line) and *T_c_* = 145 °C (solid line) (heating rate 10 K min^−1^). The thin solid lines are the solid and liquid thermodynamic specific heat capacities, as taken from the literature [45]. The inset is an enlargement of the *T_g_* region. (Reprinted (adapted) with permission from [46]. Copyright (2015) Elsevier).

As regards the mobile amorphous fraction of PLLA, a higher glass transition temperature was observed after crystallization at low *T_c_s*, and a lower glass transition temperature after crystallization at high *T_c_s* [50,51], as also shown in Figure 7. The glass transition temperature of the PLLA sample crystallized at *T_c_* = 145 °C is close to the *T_g_* of the completely amorphous PLLA, whereas the *T_g_* of the sample crystallized at *T_c_* = 85 °C is about ten degrees above. The lower glass transition temperature (*T_g,unconst_*) was connected to the devitrification of an unconstrained mobile amorphous (MA_unconstr_) fraction whereas the glass transition at higher temperature (*T_g,constr_*) was associated to the mobilization of a slightly constrained mobile amorphous (MA_constr_) fraction [38,52,53].

A detailed analysis of the evolution of the crystalline and the different amorphous fractions at different *T_c_s* (85 °C and 145 °C) proved that during crystallization at low temperature, both MA_constr_ and RA fractions develop together with the crystal phase [38]. The RA fraction vitrification occurs during the entire crystallization process, whereas the transformation from unconstrained to constrained mobile amorphous fraction seems connected with the secondary crystallization process. During crystallization at *T_c_* = 145 °C, RA fraction formation is not detected. Only upon cooling from 145 °C to room temperature, some mobile amorphous segments undergo stiffening and restriction of mobility, but with the peculiarity that only the most constrained fraction, the RA fraction, develops upon cooling, whereas the residual MA fraction remains practically unconstrained also near *T_g_*. The analysis of the evolution of the RA fraction and the different constrained MA fractions during crystallization at different temperatures and after cooling to below *T_g_*, led to assume a possible distribution of the amorphous regions [38]. The development of the RA and the MA_unconstr_ fractions in the PLLA samples crystallized at high *T_c_s* appeared not linked, probably because these fractions are placed in regions different with respect to the crystal lamellae position. Thus, it was supposed that the RA fraction develops, after crystallization at high temperature, in the inter-lamellar regions, whereas the unconstrained MA fractions remains located in the larger inter-stack gaps [38], as also demonstrated by different techniques for other polymers [54,55,56]. For crystallizations at low temperatures, which lead to α′-form growth, this type of analysis did not allow to identify univocally if RA fraction develops exclusively in the inter-lamellar regions or also at the crystal/amorphous interface [38]. 

Unlike PHB, which exhibits frequency independence of the reversing specific heat capacity when crystallized at low temperature, and PLLA, for which the frequency independence holds in a wide temperature range, for most polymers the value of *c_p,rev_* depends on the modulation frequency. In this case, *c_p,rev_* is the result of the superposition of the baseline specific heat capacity and an excess specific heat capacity, originating from the latent heat contributions of the melting and crystallization processes that occur during the two modulation semi-periods, respectively. For these polymers, isothermal crystallization is a reversing process.

**Figure 8:**
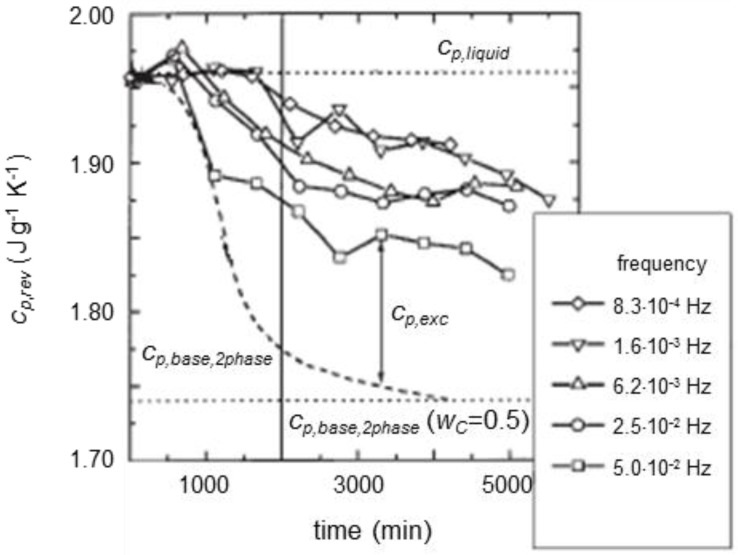
Reversing-specific heat capacity (*c_p,rev_*) during crystallization of PCL at 55 °C as a function of crystallization time (*A_T_* = 0.5 K). *c_p,base,2phase_* was calculated according to Equation (3). (Reprinted (adapted) with permission from [7]. Copyright (2000) Springer).

The dependence of *c_p,rev_* on the modulation frequency during quasi-isothermal crystallization was widely investigated for polycaprolactone (PCL) [7,24,25]. Figure 8 shows the reversing specific heat capacity of PCL during quasi-isothermal crystallization at 55 °C. The amplitude of the temperature modulation was 0.5 K, and the period ranged from 20 to 1200 s. The Figure displays the decrease in *c_p,rev_* from the value of the thermodynamic *c_p,liquid_*, in comparison with the *c_p,base,2phase_* trend calculated according to Equation (3). Through the entire crystallization process, except at the beginning, during the induction time, *c_p,rev_* remains higher than the calculated *c_p,base,2phase_* which at the end of the process corresponds to a crystalline weight fraction of 0.5. The difference between the *c_p,rev_* and *c_p,base,2phase_* is the excess specific heat capacity (*c_p,exc_*) connected to the latent heat exchanges that take place during the two modulation semi-periods.

**Figure 9:**
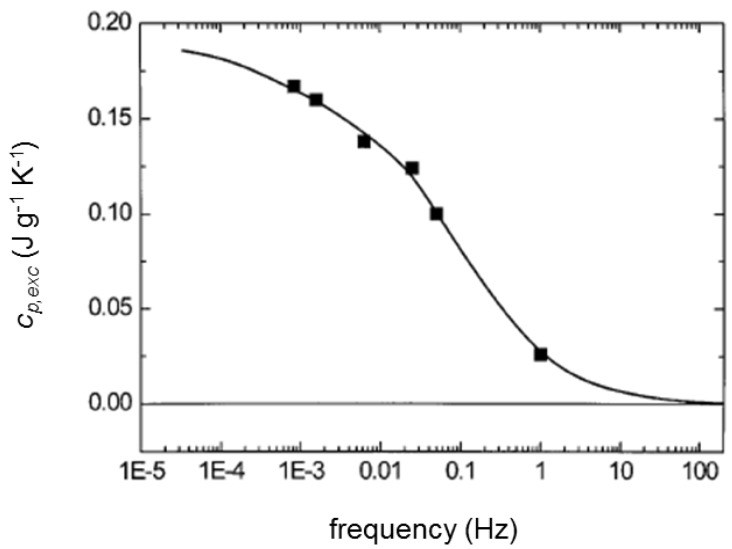
Excess specific heat capacity of PCL (*c_p,exc_*) after 2000 min of crystallization at 55 °C as a function of the modulation frequency. (Reprinted (adapted) with permission from [7]. Copyright (2000) Springer).

The frequency dependence of *c_p,exc_* for PCL, after 2000 min of crystallization at 55 °C is detailed in Figure 9: only at high modulation frequency, the reversing-specific heat capacity corresponds to the baseline capacity of the crystallization process, because the fraction of crystalline material that can follow temperature modulation decreases with increasing frequency [57,58,59]. At infinitely high modulation frequency, the reversing process is inhibited, and the baseline-specific heat capacity is generally achieved.

**Figure 10:**
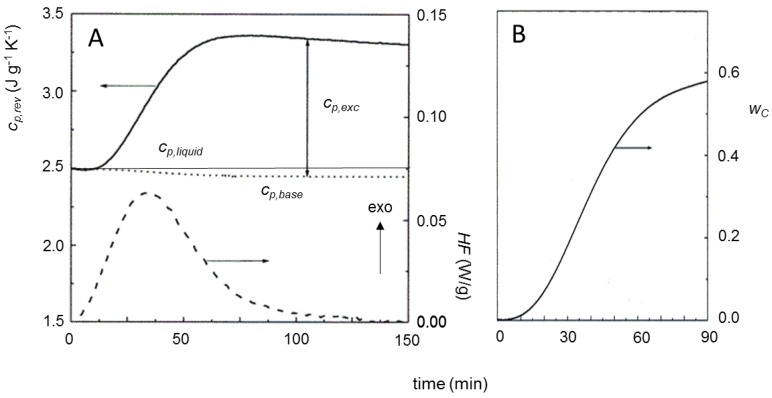
(**A**) Reversing specific heat capacity (*c_p,rev_*) of a linear polyethylene (PE) during quasi-isothermal crystallization at 126 °C (solid line). A sinusoidal temperature modulation was applied with *A*_T_ = 1.0 K and *p* = 60 s. The linear solid line is the liquid thermodynamic specific heat capacity of PE at 126 °C [63]. The *c_p,base_* curve (dotted line) was calculated according to Equation (3), on the basis of the variation of the crystalline weight fraction (*w_C_*) with time. At the bottom, *HF* (dashed line) is the average heat flow rate. (**B**) Time evolution of the crystalline weight fraction (*w_C_*), calculated as the ratio between the integrated average heat flow rate (*HF*) and the literature value for the heat of fusion of 100% crystalline polyethylene [63]. (Reprinted (adapted) with permission from [8]. Copyright (2001) American Chemical Society).

Significant *c_p,exc_* values during crystallization have been reported also for polyether ether ketone (PEEK) [24,31], PET [5,37], and polyethylene (PE) [8,9,26,60,61,62]. Figure 10 exhibits the reversing specific heat capacity curve during quasi-isothermal crystallization at 126 °C of a linear polyethylene sample [8]. The *c_p,rev_* curve progressively increases with time, up to an almost constant value, then slightly decreases, whereas the baseline specific heat capacity, as expected, decreases progressively, because the specific heat capacity of the crystalline phase is smaller than that of the amorphous phase. The trend of *c_p,rev_* is different from that reported in Figure 8 for PCL: for PE, the reversing specific heat capacity increases and remains higher than the thermodynamic *c_p,liquid_* during the whole crystallization process at 126 °C. Figure 10 also shows the evolution of the crystalline weight fraction (*w_C_*). The inflection points of the *c_p,rev_* and *w_C_* curves are located approximately at the same crystallization time, which means that the excess specific heat capacity mirrors the crystallinity degree during the initial stage of isothermal crystallization, as also reported elsewhere [61], up to completion of primary crystallization, as the inflection point roughly corresponds to the beginning of secondary crystallization [47]. 

**Figure 11:**
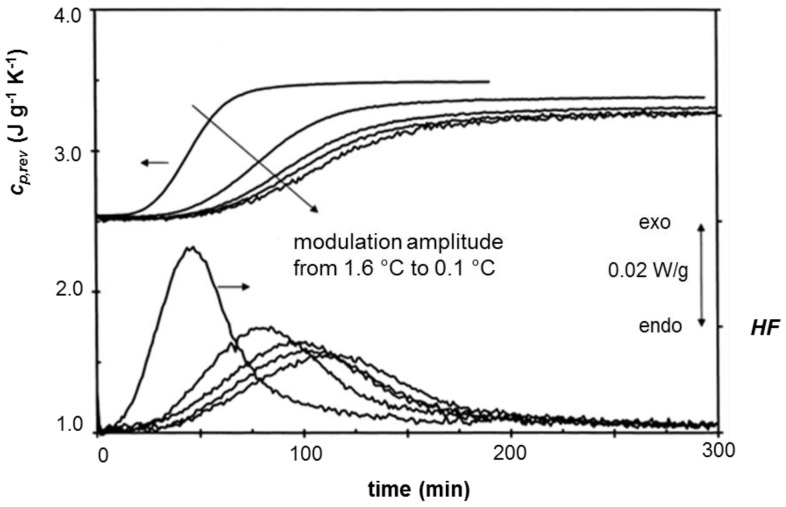
The effect of the modulation amplitude on the quasi-isothermal crystallization of linear PE at 128.5 °C: top: reversing-specific heat capacities (*c_p,rev_*) curves, bottom: average heat flow rate (*HF*) curves. (Reprinted (adapted) with permission from [26]. Copyright (1999) Elsevier).

The dependence of *c_p,rev_* on the temperature modulation amplitude is detailed in Figure 11 for quasi-isothermal crystallization of linear polyethylene at 128.5 °C. Both the reversing specific heat capacity and the heat flow rate curve shift to smaller time-scale with increasing the temperature amplitude, because of the larger temperature window covered by modulation. The result is that the crystallization rate decreases with reducing the amplitude. 

**Figure 12:**
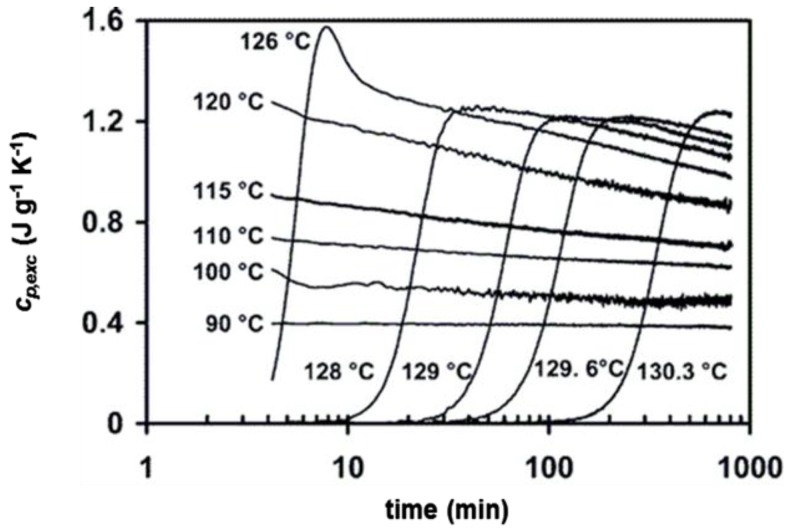
Evolution of the excess specific heat capacity (*c_p,exc_*) with time during quasi-isothermal crystallization of a linear PE at the indicated temperatures (*A_T_* = 0.8 K and *p* = 48 s). (Reprinted (adapted) with permission from [60]. Copyright (2004) American Chemical Society).

A detailed study on the connection between the excess specific heat capacity and the evolution of the crystallinity degree and melting temperature during isothermal crystallization of a linear PE was conducted by Marand et al. [60]. The *c_p,exc_* curves determined during quasi-isothermal crystallizations in a wide temperature range are shown in Figure 12. 

**Figure 13:**
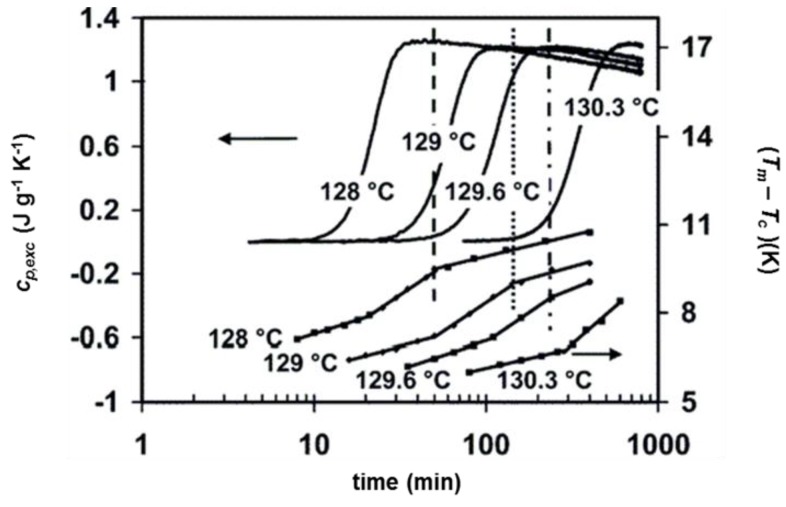
Correspondence between the three stages of the melting temperature (*T_m_*) evolution, plotted as the difference between the melting temperature and the crystallization temperature (*T_c_*), and the decay of the excess heat capacity at the indicated crystallization temperature. (Reprinted (adapted) with permission from [60]. Copyright (2004) American Chemical Society).

At high crystallization temperature, as also shown in Figure 10, the excess specific heat capacity increases during the initial stage of the process, and after reaching a maximum, decays slowly. At lower temperatures, when primary crystallization is completed during the previous cooling step, only the decay is observed (Figure 12). The peak melting temperature was found to shift to higher values with increasing the crystallization time (Figure 13). The time evolution of the melting temperature had a sigmoidal shape, thus the entire crystallization process was roughly divided into three stages: the first and third stages were put into relation with pure primary and secondary crystallization processes respectively, whereas the middle step was associated to a mixed regime of primary and secondary crystallization. Figure 13 exhibits the correlation between the evolution of the excess specific heat capacity during quasi-isothermal crystallization at high temperatures and the corresponding melting temperature progression. The onset of the decay of the excess specific heat capacity was found to correspond approximately to the beginning of the third stage of the melting temperature evolution, i.e., to the secondary crystallization event, which in PE takes place mainly through lamellar thickening [64]. This process in PE is connected to a crystal α_c_ relaxation process, which involves cooperative chain sliding in the crystalline regions [65]. The decay of the excess specific heat capacity in PE at high crystallization temperature was thus connected with lamellar thickening, and therefore associated with molecular motions occurring in the fold interface regions [60]. Conversely, reversing crystallization/melting that takes place in PE at low temperatures was attributed to lateral crystal surface activity, based on partial melting and crystallization without the need of molecular nucleation, according to the mechanism typical of polymers that do not exhibit sliding diffusion [66,67].

To summarize, for some polymers, for example PCL and PE, the dynamics of the reversing melting is fast, and, as a consequence, considerable *c_p,exc_* values are measured and *c_p,rev_* might correspond to the baseline specific heat capacity only if the modulation period is very short. When, on the contrary, the dynamics of the reversing melting is sufficiently slow, for example for PHB at low crystallization temperature, or for PLLA, the high-frequency limit is reached at standard frequencies of TMDSC, and *c_p,rev_* provides the thermodynamic baseline specific heat capacity during the entire the crystallization process. 

**Figure 14:**
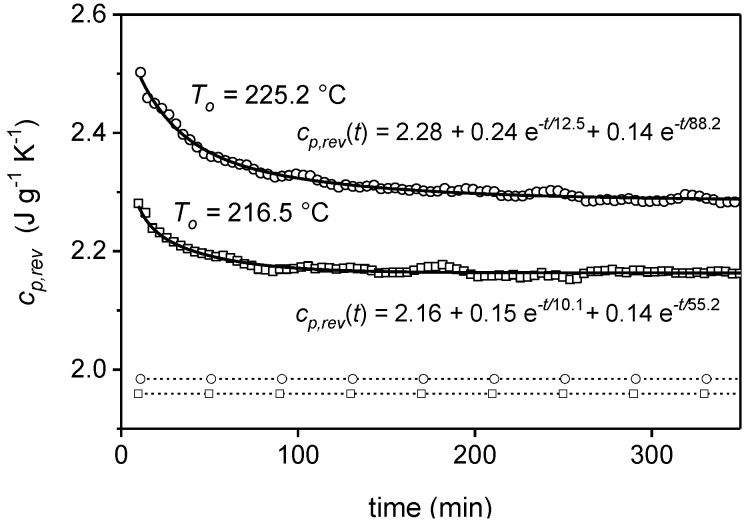
Time dependence of the reversing specific heat capacity (*c_p,rev_*) of PBT isothermally crystallized at 200 °C for 30 min, during quasi-isothermal measurements over long time periods in the melting region at *T_o_* = 216.5 °C (□) and 225.2 °C (○) (*p* = 120 s, *A_T_* = 0.2 K). The dashed lines with squares and circles are the *c_p,base,2phase_* values calculated by Equation (3) from the crystallinity degrees measured at the end of the quasi-isothermal annealing at *T_o_* = 216.5 °C and 225.2 °C, respectively, by using the solid and liquid specific heat capacities of PBT as taken from [40]. (Reprinted (adapted) with permission from [73]. Copyright (2004) American Chemical Society).

The study of the time dependence of the reversing specific heat capacity during quasi-isothermal experiments has demonstrated that when *c_p,exc_* is not zero, *c_p,rev_* can tend to a constant value, higher than the expected thermodynamic *c_p,base_*. This condition identifies a reversible melting process, i.e., the occurrence of a true local equilibrium at the crystal/amorphous interface [2]. The phenomenon has been studied mainly in the melting region of various polymers, for example PET, poly(butylene terephthalate) (PBT), poly(trimethylene terephthalate) (PTT), PS, nylon12, *cis*-1,4-polybutadiene [68,69,70,71,72,73,74,75]. During a quasi-isothermal annealing around a given temperature (*T_o_*) in the melting region, *c_p,rev_* decreases progressively towards a constant value, as illustrated in Figure 14 for a semi-crystalline PBT sample [73]. These relaxation curves are generally fitted with a sum of two exponential decay functions [2]:

(8) $$c_{p,rev}\left(t\right)=c_{p,\infty }+C_{1}e^{-t/\tau_{1}}+C_{2}e^{-t/\tau_{2}}$$

where $c_{p,\infty }$ is the final equilibrium reversible specific heat capacity, and $\tau_{1}$ and $\tau_{2}$ the relaxation times associated to irreversible events that occur during the quasi-isothermal analysis, as partial melting and fast recrystallization, which are connected with the lower relaxation time, and slow crystal perfection, related to the higher relaxation time [71,72]. Partial melting leads to the removal of fully melted molecules from the reversing melting, because these molecules would need a new molecular nucleation to recrystallize. Recrystallization and crystal perfection also produce a reduction in *c_p,rev_*, because more perfect crystals, with higher melting temperature, are removed from the reversing cycle. After long times, all these irreversible events disappear, and a steady state is reached, which corresponds to a situation of local equilibrium. In Figure 14, the occurrence of a true reversible local equilibrium at the crystal/amorphous interface is attested by the final *c_p,rev_* values, which are higher than the corresponding *c_p,base,2phase_* data calculated by Equation (3) from the values of the crystallinity degrees measured at the end of the quasi-isothermal measurements [73].

**Figure 15:**
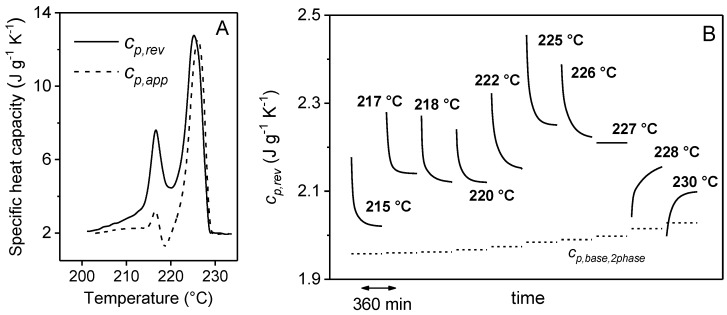
(**A**) Apparent specific heat capacity (*c_p,app_*, dashed line) and reversing specific heat capacity (*c_p,rev_*, solid line) of PBT isothermally crystallized at 200 °C for 30 min as a function of temperature during heating (heating rate: 0.5 K min^−1^, modulation period: 120 s, temperature modulation amplitude: 0.2 K); (**B**) time dependence of the reversing specific heat capacity (*c_p,rev_*) of PBT during quasi-isothermal measurements of 6 h at the indicted *T_o_s*. The data are from separate measurements and are collected in a single graph in order to compare the *c_p,rev_* trend at different temperatures. The dashed lines are the *c_p,base,2phase_* values calculated by Equation (3) from the values of the crystallinity degrees measured at the end of the quasi-isothermal annealing, by using the solid and liquid specific heat capacities of PBT as taken from [40]. (Reprinted (adapted) with permission from [73]. Copyright (2004) American Chemical Society).

The time evolution of the reversing specific heat capacity during quasi-isothermal annealing depends on *T_o_*. Figure 15 illustrates the results of quasi-isothermal analyses performed for 6 h at different *T_o_s*, chosen in the melting range of a PBT sample isothermally crystallized at 200 °C [73]. The *c_p,app_* and *c_p,rev_* curves registered immediately after crystallization upon heating at 0.5 K min^−1^, are shown in the graph on the left. For *T_o_* < 227 °C, the *c_p,rev_* curves registered during the quasi-isothermal annealing (plot on the right) display the common observed decay. When PBT is analyzed at 227 °C, the reversing specific heat capacity remains practically constant during the entire experiment. For quasi-isothermal analyses performed at higher temperatures (228 °C and 230 °C), *c_p,rev_* shows a different trend, increasing with the measurement time. These last experiments start from an almost completely melted sample, which partially recrystallizes during the quasi-isothermal measurements. This recrystallization progressively add new crystals to the reversing melting process: the effect is a continuous increase in the measured *c_p,rev_*. At the intermediate temperature of 227 °C, fusion, recrystallization and crystal perfection are clearly counterbalanced, which results in a constant *c_p,rev_* value. Also, in Figure 15, the comparison between the *c_p,rev_* values at the end of the quasi-isothermal annealing and the corresponding calculated *c_p,base,2phase_* data prove that a local reversible melting/crystallization process is established in PBT after long times of annealing. 

## 4. Non-Isothermal Crystallization and Stepwise Quasi-Isothermal Crystallization upon Cooling

Non-isothermal crystallization that takes place upon cooling from the melt, sometimes named ‘hot crystallization’, usually exhibits a reversing response in TMDSC analyses. The amount of the reversing melting generally increases with increasing temperature, and it is higher in the melting region with respect to the crystallization region [2]. At the low average cooling rates used with the TMDSC technique (generally 1–2 K min^−1^), non-isothermal crystallizations occur at temperature far above the glass transition temperature. For this reason, the excess specific heat capacity largely contributes to *c_p,rev_*, with the result that it is impossible to attain the baseline specific heat capacity of the non-isothermal crystallization by TMDSC, as proven, for example, in the case of PET and PHB [36,37].

**Figure 16:**
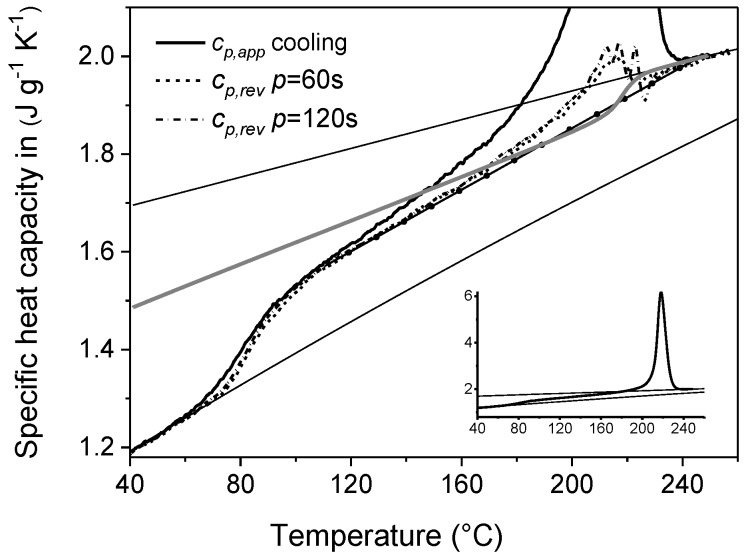
Apparent specific heat capacity (*c_p,app_*) by standard DSC and reversing specific heat capacity (*c_p,rev_*) of PET at modulation period of 60 s and 120 s, measured upon cooling from the melt at 2 K min^−1^ (*A_T_* = 1.0 K). The black solid line with circles is the approximate linear baseline, whereas the solid grey line is the two-phase baseline (*c_p,base,2phase_*). The black thin solid lines are the solid and liquid specific heat capacities of PET, as taken from [40]. The inset shows the entire *c_p,app_* curve. (Reprinted (adapted) with permission from [37]. Copyright (2014) Elsevier).

An example of non-isothermal crystallization monitored during TMDSC cooling run is shown in Figure 16. The apparent specific heat capacity (*c_p,app_*) of PET measured by conventional DSC at cooling rate of 2 K min^−1^, is compared in Figure 16 with the corresponding *c_p,rev_* curves by TMDSC with *p* = 60 s and 120 s (average cooling rate: 2 K min^−1^). On the same graph, the specific heat capacity data in the liquid and solid state of PET, as taken from the literature, are also indicated. 

The *c_p,app_* curve depicted in Figure 16 reveals that the crystallization process extends from about 240 °C down to approximately 120 °C, with the peak centered at 218 °C. The glass transition, related to vitrification of the MA fraction, is centered approximately at 80 °C. At temperatures below 140 °C, the overlapping of the *c_p,rev_* curves, which are independent of the frequency of modulation, attests that no reversing latent heat is exchanged in this temperature range upon cooling. As a result, between approximately 140 °C and the glass transition temperature, the *c_p,rev_* curves correspond to *c_p,base_* of the crystallization process. Conversely, at temperatures higher than 140 °C, the *c_p,rev_* curves do not define the baseline specific heat capacity, because they exhibit a peak that originates from reversing crystallization/melting or change in the crystallization rate between the two semi-periods. The fast release of latent heat that occurs during crystallization can cause false effects in the calculated reversing specific heat curve [76]. The irregular shape of the *c_p,rev_* curves, which becomes more wrinkled in the crystallization temperature range with increasing the modulation period, is an example of these possible artifacts. As a first approximation, a baseline-specific heat capacity can be constructed by extrapolating up to melt the linear trend displayed from 100 °C to about 140 °C by the *c_p,rev_* curves. The linear baseline allows the *c_p,app_* curve to be integrated, to determine the crystalline weight fraction evolution, according to Equation (9) [37]:

(9) $$w_{C}(T)=\int_{T}^{T_{o}}\frac{c_{p,app}(T\prime )-c_{p,base}(T\prime )}{\Delta h_{m}^{o}(T\prime )}dT\prime$$

where *T_o_* is a reference temperature in the melt.

**Figure 17:**
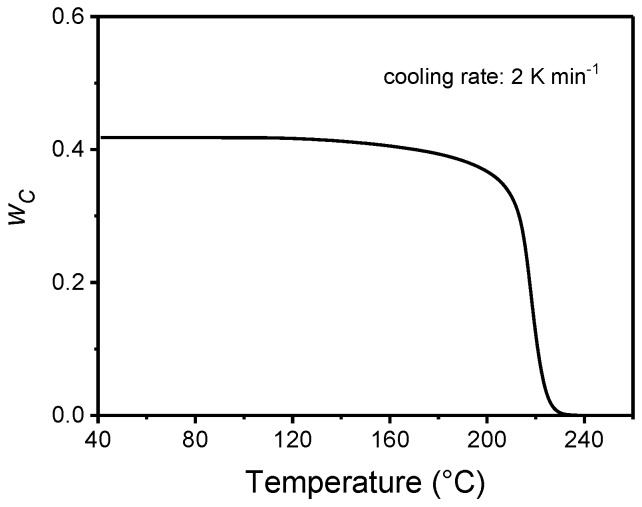
Temperature evolution of the crystalline weight fraction (*w_C_*) of PET during non-isothermal crystallization at 2 K min^−1^. (Reprinted (adapted) with permission from [37]. Copyright (2014) Elsevier).

The calculated temperature-dependence of the crystalline weigh fraction *w_C_* is presented in Figure 17. The final *w_C_* is 0.42, a value quite high due to the slow cooling rate used in the TMDSC measurements [77,78]. From this *w_C_* curve, a two-phase approximate baseline specific heat capacity (*c_p,base,_*_2*phase*_), which neglects vitrification of the rigid amorphous fraction, can be determined, according to Equation (3). Figure 16 shows that *c_p,base,_*_2*phase*_ crosses the approximate linear baseline around 190 °C. This finding suggests that part of the amorphous chains might start to vitrify during crystallization at 2 K min^−1^ approximately at 190 °C. A more correct sigmoidal *c_p,base_* curve can be constructed by merging the *c_p,base,_*_2*phase*_ curve from the beginning of the crystallization to 190 °C, to the linear *c_p,base_* curve from 190 °C down to the glassy state. The resultant *w_C_*, determined by Equation (9) using the new baseline, is practically coincident with that determined with the linear baseline (∆*w_C_* = 0.001 at 40 °C). This type of analysis, which combines conventional and temperature-modulated DSC measurements, can thus allow an approximate identification of the temperature at which the RA fraction starts to develop during non-isothermal crystallization. 

**Figure 18:**
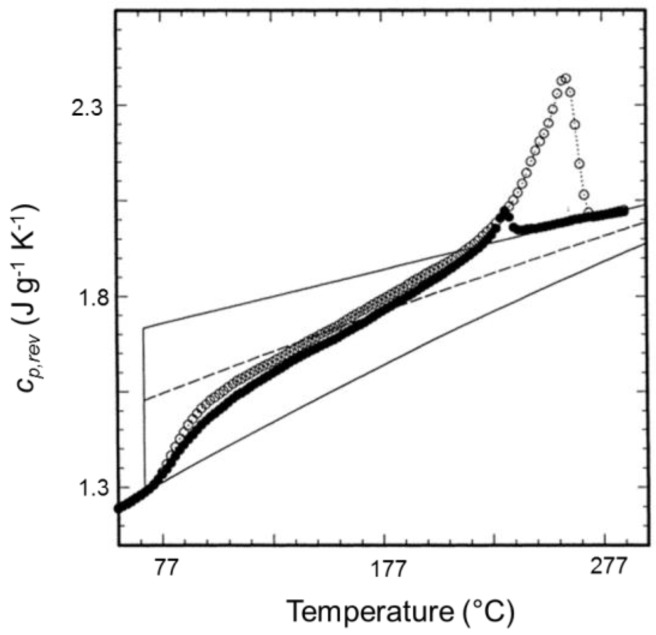
Reversing-specific heat capacity (*c_p,rev_*) of PET (filled circles) during stepwise quasi-isothermal TMDSC cooling from the melt (*p* = 60 s and *A_T_* = 1.0 K, oscillation time around each temperature: 20 min). The thin solid lines are the solid and liquid specific heat capacities, as taken from [40]. The broken line is the calculated specific heat capacity for a 49% crystalline PET. The open circles describe the *c_p,rev_* curve of a melt-crystallized PET sample (crystallinity degree: 44%). (Reprinted (adapted) with permission from [5]. Copyright (1997) American Chemical Society).

Similar *c_p,rev_* curves were registered for PET during stepwise quasi-isothermal cooling from the melt (see Figure 18). After crystallization, the crystallinity percentage, determined on re-heating by standard DSC, was 0.49, a value slightly higher than the final *w_C_* determined at the end of the experiment described in Figure 16 and Figure 17. The reason is ascribable to the much smaller average cooling rate of the stepwise quasi-isothermal run (0.1 K min^−1^). For a comparison, Figure 18 shows also the *c_p,rev_* curve of a semicrystalline PET sample with similar crystallinity degree, obtained through a stepwise quasi-isothermal heating experimental procedure.

**Figure 19:**
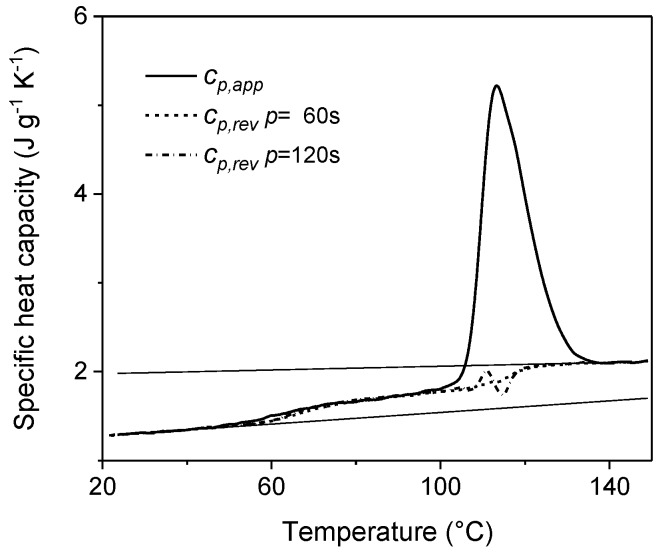
Apparent specific heat capacity (*c_p,app_*: solid lines) of PLLA and reversing specific heat capacity (*c_p,rev_*) on cooling at 2 K min^−1^ as a function of temperature (*p* = 60 s: dashed line; *p* = 120 s: dashed dotted line). The thin solid lines are the thermodynamic solid and liquid specific heat capacities of PLLA as taken from the literature [45]. (Reprinted (adapted) with permission from [79]. Copyright (2017) Springer).

Up to now, only the reversing specific heat capacity of PLLA has been found to be independent of frequency modulation during non-isothermal crystallization from the melt. Figure 19 shows the *c_,app_* curve of a PLLA sample obtained by conventional DSC during cooling from the melt at 2 K min^−1^ together with the corresponding *c_p,rev_* curves by temperature-modulated DSC (average cooling rate: 2 K min^−1^, temperature amplitude: 1.0 K, modulation periods: *p* = 60 s and 120 s, respectively) [79]. The crystallization peak extends from about 130 °C to approximately 90 °C, and appears slightly asymmetric. This irregular shape, already reported in the literature [80], has been attributed to the growth of the two different crystal forms, the α-form at higher temperatures, and the α′-form at lower temperatures [42,43,44]. In all the samples, the reversing specific heat capacity upon cooling appears frequency independent (within the experimental error and noise) in the whole crystallization range, which means that reversing latent heat is not exchanged during the temperature modulation and that the *c_p,rev_* curves correspond to the baselines of the crystallization process.

The reduction in the MA fraction during non-isothermal crystallization was derived according to Equation (7), and the growth of the crystalline weight fraction from the integration of the crystallization peak, according to Equation (9), by using for $\Delta h_{m}^{o}\left(T\right)$the average of the enthalpy of fusions of the 100% crystalline α′- and α-forms [46]. Finally, the RA fraction evolution upon non-isothermal crystallization was determined by difference. 

**Figure 20:**
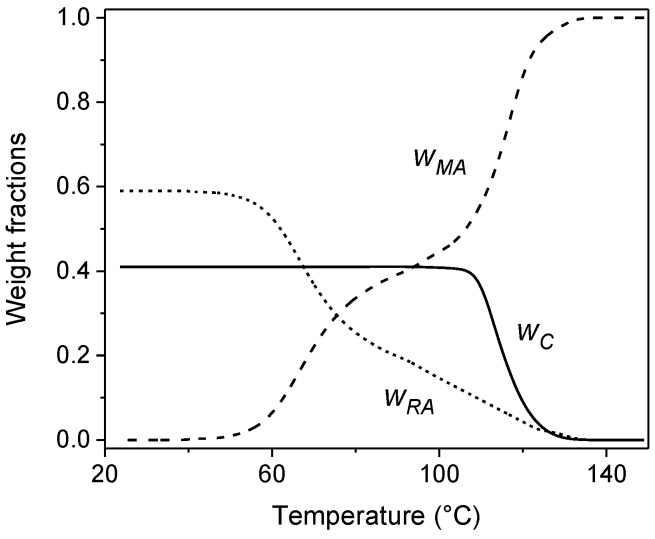
Temperature evolution of the mobile amorphous (*w_MA_*: dashed line), crystalline (*w_C_*: solid line), and rigid amorphous (*w_RA_*: dotted line) weight fractions during non-isothermal crystallization of PLLA at 2 K min^−1^. Estimated errors: ±0.02 for *w*_C_ and *w_MA_*, ±0.04 for *w_RA_*. (Reprinted (adapted) with permission from [79]. Copyright (2017) Springer).

Figure 20 displays the temperature evolution of crystalline (*w_C_*), mobile amorphous (*w_MA_*) and rigid amorphous (*w_RA_*) weight fractions during non-isothermal crystallization of PLLA at 2 K min^−1^. During the non-isothermal crystallization, simultaneously with the increase in *w_C_*, the MA fraction decreases progressively, as evidenced by the marked first step in the temperature range 130 °C ÷ 100 °C. A second step is observed in the *w_MA_* curve in the glass transition region between 80 °C and 60 °C, when all mobile amorphous fraction becomes vitrified.

The RA fraction develops in parallel with the crystal phase, starting from approximately 130 °C. This further validates the hypothesis that 130 °C is likely the upper limit temperature for the RA fraction in PLLA [34,38]. The development of the rigid amorphous fraction occurs simultaneously with crystal growth, both during primary and secondary crystallizations. Between the apparent end of the crystallization, approximately 90 °C, and the beginning of the glass transition, the RA fraction continues to increase. This could be due to a minor and undetectable increase in *w_C_*, which, occurring in geometrically restricted areas, could induce stiffening of an additional fraction of amorphous segments. Otherwise, since RA fraction originates from amorphous segments that are constrained above *T_g_*, it is probable that internal stresses, which are not released during crystal growth, and are concentrated at the amorphous/crystal interface, can produce some amorphous segments vitrification upon cooling, as a consequence of the reduced mobility of the chains [81]. In addition, rigid crystalline domains could produce, through a friction or “wall” effect, an elevation of the glass transition temperature of the amorphous segments located in proximity of the rigid crystal surfaces [82,83]. The final increase in the RA fraction, observed at *T_g_*, reflects vitrification of the MA fraction, because at temperatures lower than *T_g_*, all the material is solid.

## 5. Conclusions

At the end of this review, it is worth emphasizing further that the combination of conventional DSC and TMDSC is a useful path that can allow the obtainment of much information on the crystallization and thermal behavior of polymeric materials. Although TMDSC measurements are slow and quite time-consuming, the calorimetric data that can be derived are crucial for a more detailed description of the thermal properties of polymeric materials. By means of the temperature-modulated calorimetric technique, evolution of the baseline specific heat capacity can be assessed during polymer crystallization, which makes the calculation of the crystallinity degree much more accurate. This possibility is offered especially at low crystallization temperatures, when the amount of reversing melting is smaller. Particularly interesting is the case of PLLA, which does not exhibit dependence of the reversing specific heat capacity on the modulation frequency, so that crystallization occurs irreversibly in a wide temperature range. This favorable situation has allowed the accurate determination of the evolution of the crystalline, mobile amorphous and rigid amorphous fractions during solidification in different experimental conditions, and has allowed the distribution of the amorphous regions with respect to the crystalline areas to be supposed. The description of the micro- and nano-phase structure of PLLA was thus attained. This type of investigation, detailed in this review in particular for PLLA, is a procedure advantageous and very useful for the assessment and prediction of many physical characteristics, because it has been proven that many properties of polymeric materials, for example mechanical and gas permeability, depend not only on the crystallinity, but also on the rigid amorphous fraction, and on its evolution during solidification [84,85,86,87,88,89,90,91].
